# The Etiology of Caries of the Teeth Viewed from the Standpoint of Physiological Chemistry

**Published:** 1888-02

**Authors:** L. G. Noel

**Affiliations:** Nashville, Tenn.


ARTICLE VI.

### THE ETIOLOGY OF CARIES OF THE TEETH VIEWED FROM THE STANDPOINT OF PHYSIOLOGICAL CHEMISTRY.

L. G. NOEL, M. D., D. D. S., NASHVILLE, TENN.

[Read before the Southern Dental Association, Old Point Comfort, Va., September, 1887.

The most important topic to be considered by your Committee is the causes of caries of the teeth. This question involves all that is known of the chemistry of the fluids of the mouth and alimentary canal and of their physiological action. Its importance commands the most earnest attention, the most indefatigable zeal and profoundest research of the dental profession. Whilst we deplore the paucity of our knowledge, we may congratulate ourselves that so many brilliant minds are engaged in the work of investigation, and look forward to the dawn of a brighter day.

We shall not consume your time in recalling to your minds the researches of the advocates of the germ or fermentation theory, with the various arguments they adduce to sustain it. Nor shall we repeat here, all that has been said in rebuttal. Some recent consideration of the subject on our own part, has led us to modify our preconceived notions, and brought us to concede that the truth lies between. Some one has said, "there is a soul of truth in all error." It must needs be so. The skeleton or framework of truth, must be there, and how spaciously we built on muscles, tendons, integuments and clothe our manikin in all the beauty of reality. If we can but show you this framework of truth in the germ theory of caries, and convince you of its existencc, we shall be content. Nor does the acceptance of it compel the relinquishment of other doctrines as ably sustained in the past; they too may be found to be builded upon a framework as enduring and strong. The labors of such earnest investigators as Dr. Geo. Watt, have not been in vain. His advocacy of the theory of the origin and action of the mineral acids in the mouth, and his clear showing of the peculiar foot-print of each, has never been refuted. We think the labors of Magitot, Miller and Watt, may be harmonized and combined into a grand whole, that shall lead us into the sunlight of the glorious day of certainty, but much labor and much patient effort lies between us and the goal. Now why should I tax your patience when I can so quickly lead you to the gist of the matter.

We have only to lift up our heads and take our eyes out of the mouth for a moment—take a little wider and more comprehensive view of nature's processes—forget we are dentists for a time, and become students of nature, to see this thing. It is a well known fact, that oftentimes, chemical agents whose affimities would lead them to combine with each other, will lie in contact for the longest time without noticing each other; they behave more like distant and reserved people at a watering place, or seashore resort, than any thing we can liken them to. Well, what is the matter. They are only in need of a third party to introduce them to each other, and when that ceremony is performed, they buzz and hum like a bee-hive, until presto, they are pairing off and getting married. We come now to the heart of the nut. The whole story may be told in one word, and that one word is Catalysis ! We have an example of this action in the diastase of fruit, which has the power of converting the starch of the fruit into glucose. We have a similar ferment ptyaline, in saliva, which produces a similar effect upon starchy foods, the glucose thus formed greatly facilitating deglutition and the function of taste. Then again, pepsin is the digestive ferment of the stomach by which albuminous matter is transformed into peptone. This ferment acts upon the food in the presence of an acid, and experiments have shown that the acid may be changed from hydro-chloric to lactic, and still the digestion of the food go on, if the pepsin be present in sufficient quantity. Its action like that of ptyaline in the saliva, is a purely catalytic one. Caries of the teeth may be likeened to the digestive process. We have the lodgement of foods in the fissures, interstices, and about the gum borders on the buccal and labial surfaces of the teeth—foods capable of being transformed by the ferments and acids having access to them. The lichen like fungi, described by our microscopists as found in carious cavities, has numerous analogues elsewhere in nature, as for example, the fungi of yeast and of malt. It appears to be only a growth of the catalytic substance and whilst it does not exert a direct action itself, upon the tissucs, it is the third party bringing about the mischief, by the ceremony of introduction. Take the transformation of starchy particles under consideration for a moment. Cooked starch is changed into glucose by the ptyaline of the saliva, in one minute. Glucose is the most sensitive of all saccharine substances to ferments. We can readily follow its further changes into acetic and lactic acids, and thus account for much destructive action upon the teeth. Ptyaline is powerless to produce glucose from raw starch, and it has been proven by experiments that no glucose is to be found in the mouths of herbivorous animals, accustomed to take their food raw. Nor do we find caries of the teeth, in the mouths of such animals. May this not account in some measure for the freedom from decay in the case of savages, whose food is generally taken in the raw state? We find a similar ferment in the pancreati juice—pancreatin. Dalton calls this a diastatic ferment. Its action upon starch is much more rapid and more thorough than that of the saliva converting the whole mass rapidly into a soluble substance capable of immediate absorption. The pancreatic ferment also operates through the medium of an abid menstrum, and this acid is thought to be lactic, but it may undergo changes, as similar results have been obtained experimentally, with different mild acid solutions containing pancreatin. The pancreatic fluid possesses far greater potency for transforming starch into glucose than the saliva, converting large masses in a short time and has this power to a certain extent with raw starch as is shown by the digestion of raw starch, by the herbivora. It has other functions equally important, for it rapidly emulcifies and renders soluble the fatty matters, enabling them to get into the blood through the lacteals.

Note then the potency of acids to dissolve substances of various kinds in the presence of these catalytic agents, of which we know so little—the ferments. Note also the analogy here. We have in all these digestive or solvent processes, mild acid fluids containing a ferment. Now is it difficult to see the analogy to what occurs in the interstices between the teeth, and in the fissures of the enamel, and about the borders of the gum, anywhere in fact that food may lodge about teeth and undergo decomposition? It must be remembered that these ferments have that leavening power spoken of in the scriptures: "A little leaven leavenith the whole lump." Once let them get established in the mouth and it is easy to account for their action upon all alimentary substances that may be detained there long enough to undergo change. Is it difficult to conceive of these mild acids acting upon the enamel in places of habitual lodgements, and the ultimate formation of cavities of decay, with all the attendant phenomena of fungi, and need we throw out of the count such argument—such *proof* as Watt adduces of the origin of the mineral acids from decomposing alimentary substances. We know it will be said these views are not proof, they are mere reasonings, mere hypotheses Gentlemen, this is all we claim for them, and if as such you deem them worthy of your consideration, we are content. Prof. Tyndall in his "Fragments of Science," has published an eloquent chapter on the importance of indulging and cultivating the powers of the imagination. "Without its aid, he says, we can never make progress in discovery, it must go before and blaze out through the wilderness of the unknown a pathway for the discoverer." This dim and indistinct outline will serve him as a tracer for the location of the great highway. The astronomer must first project his fancy far into the illimitable realms of space. He must first imagine the existence of a planet before he can discover and disclose it. This, gentlemen, by way of apology for these crude analogies I bring before you. Now a word as to application. You are so eminently practical you have already asked the question: "Well, what are we going to do to save our teeth from being dissolved out of our mouths, if you are going to make us digest our own teeth?" Gentlemen, why do we not digest our own stomachs and pancreas? Simply because we keep up during life too much resistling vitality. Digestion of the stomach by its own juices actually has been known to occur *after death*, when the pepsin was present in large quantity. Teeth possess but feeble vitality but this is all that stands between them and destruction. That the teeth of the ancient Gaul were better than are those of the modern Englishman or Frenchman, and that those of the North American Indian in his savage state were better than those of the civilized inhabitants of this country to-day, is generally conceded. The reason for this is also conceded as due to the simplicity of savage life. The constant use of the teeth in masticating primitively prepared foods, the abundant nutrition furnished the osseous structures in such foods, and the beneficent effects of active outdoor exercise. Savage dentures were subjected to many of the same destructive agents that prey upon the teeth of civilized races, but they were enabled to run the gauntlet, protected by better inherent structure, and provided with a more enduring vitality. We have but to furnish sufficient and properly prepared aliment, restore to our teeth their lost function, observe proper hygiene habits and we verily believe that a race surrounded by a jealous and strict environment that would preclude the possibility of intermarriage with outside parties, might be developed up into a people possessing ideal skeletons and denture.—*Southern Dental Journal.*

---

ARTICLE VII.

